# Understanding the pH Dependence of Supersaturation State—A Case Study of Telmisartan

**DOI:** 10.3390/pharmaceutics14081635

**Published:** 2022-08-05

**Authors:** Szabina Kádár, Dóra Csicsák, Petra Tőzsér, Attila Farkas, Tamás Pálla, Arash Mirzahosseini, Blanka Tóth, Gergő Tóth, Béla Fiser, Péter Horváth, János Madarász, Alex Avdeef, Krisztina Takács-Novák, Bálint Sinkó, Enikő Borbás, Gergely Völgyi

**Affiliations:** 1 Department of Organic Chemistry and Technology, Budapest University of Technology and Economics, 3 Műegyetem rkp., 1111 Budapest, Hungary; 2 Department of Pharmaceutical Chemistry, Semmelweis University, 9 Hőgyes Endre Street, 1092 Budapest, Hungary; 3 Department of Inorganic and Analytical Chemistry, Budapest University of Technology and Economics, Műegyetem rkp. 3, 1111 Budapest, Hungary; 4 Institute of Chemistry, University of Miskolc, Miskolc-Egyetemváros, 3515 Miskolc, Hungary; 5 Ferenc Rákóczi II Transcarpathian Hungarian College of Higher Education, 90200 Berehove, Ukraine; 6 In-ADME Research, 1732 First Ave., #102, New York, NY 10128, USA; 7 Pion Inc., 10 Cook Street, Billerica, MA 01821, USA

**Keywords:** supersaturation, telmisartan, solubility, dimerization

## Abstract

Creating supersaturating drug delivery systems to overcome the poor aqueous solubility of active ingredients became a frequent choice for formulation scientists. Supersaturation as a solution phenomenon is, however, still challenging to understand, and therefore many recent publications focus on this topic. This work aimed to investigate and better understand the pH dependence of supersaturation of telmisartan (TEL) at a molecular level and find a connection between the physicochemical properties of the active pharmaceutical ingredient (API) and the ability to form supersaturated solutions of the API. Therefore, the main focus of the work was the pH-dependent thermodynamic and kinetic solubility of the model API, TEL. Based on kinetic solubility results, TEL was observed to form a supersaturated solution only in the pH range 3–8. The experimental thermodynamic solubility-pH profile shows a slight deviation from the theoretical Henderson–Hasselbalch curve, which indicates the presence of zwitterionic aggregates in the solution. Based on p*K*_a_ values and the refined solubility constants and distribution of macrospecies, the pH range where high supersaturation-capacity is observed is the same where the zwitterionic form of TEL is present. The existence of zwitterionic aggregation was confirmed experimentally in the pH range of 3 to 8 by mass spectrometry.

## 1. Introduction

Achieving supersaturation in solution is highly desired in the case of poorly water-soluble active pharmaceutical ingredients (API) belonging to the Biopharmaceutical Classification System (BCS) classes II and IV. Although the number of publications on developing formulations to achieve a supersaturated state during dissolution is rapidly increasing [1,2,3], supersaturation as a solution phenomenon is still challenging to understand, and therefore many recent publications focus on this topic [4,5,6,7,8]. While most studies focus on finding the upper limit of supersaturation called amorphous or kinetic solubility with state-of-the-art analytical techniques [9,10,11,12,13], only a little information is available about the molecular interactions or structural and physicochemical properties that may enable a drug in solution to reach supersaturation. The fact that the pH of the gastrointestinal environment is dynamically changing from acidic to neutral, adds to the complexity of supersaturation phenomenon, since pH dependence in species associations needs to be also considered.

The knowledge of p*K*_a_ values is required to study the pH dependence of solubility and supersaturation but determining the constants in aqueous media of BCS II and IV drugs is challenging on its own because of their low water solubility. Multiple methods, like potentiometric, UV-pH, or NMR-pH titration are available for p*K*_a_ determination [14,15,16].

A widely used and validated technique for measuring the equilibrium solubility is the saturation shake-flask (SSF) method, which is performed in the presence of an excess of the solid substance [17]. Solid-state characterizations must also be performed at the end of the measurement, since in the presence of a solvent changes in the structure of the solid compound may influence the measurement results (salt formation, polymorphic transformation, crystallization) [18]. Differential scanning calorimetry (DSC), X-ray diffraction (XRD), and Raman spectroscopy are the most commonly used techniques for solid-state analysis [19,20,21]. One of the main disadvantages of the SSF method is that it requires phase separation, which can be performed by sedimentation, centrifugation, or filtration. For example, the results of the solubility measurement using filtration—the most frequent phase separation method in the pharmaceutical industry—can be misleading due to the interactions between the filter material and the API in the solution [22]. Sedimentation and centrifugation techniques also have drawbacks, especially in case of compounds with low stability. In recent years, methods without phase separation have been available, using in situ concentration determination with UV probes, which may minimize some of the possible uncertainties [19].

Using the p*K*_a_ and intrinsic solubility value, the theoretical solubility-pH profile of an API can be predicted by the Henderson–Hasselbalch (HH) equation. However, the measured solubility values frequently show deviation from the theoretical values because of salt or micelle formation and aggregation, as well as complexation with buffer constituents [23,24,25,26,27]. According to the literature, mass spectrometry may be a suitable method to identify aggregation as a cause for the deviation from the HH curve [25].

For reaching supersaturation, the thermodynamic solubility of the API must be exceeded in solution. The upper concentration limit of supersaturation can be defined with kinetic solubility, the concentration in solution when an induced precipitate first appears. If the precipitating solid phase is analyzed and proven to be amorphous, only then the result can be called ‘amorphous solubility [28]. Supersaturation may be reached in several ways: cosolvent quench method [18,29], pH-shift method [10], or a stock solution can be prepared by raising the temperature resulting in saturated hot solution.

This work aimed to investigate and better understand the pH-dependence of supersaturation of telmisartan (TEL) at a molecular level and find a connection between physicochemical properties of API and the ability to form a supersaturated solution of TEL. Therefore, the main focus of the work was on the pH-dependent thermodynamic and kinetic solubility of the model API, TEL. Additionally, dissociation constant determinations were performed to complete physicochemical profiling of TEL.

TEL is a BCS II drug, and is a widely used antihypertensive agent that belongs to the angiotensin II receptor blockers group [30]. TEL has three ionizable groups; therefore, its solubility is pH-dependent and the compound has low solubility between pH 3–8 [31,32]. In the literature, the p*K*_a_ data of TEL are inconsistent [33,34] and the reported solubility data show huge deviations [35,36].

## 2. Materials and Methods

### 2.1. Materials

Buffer components (KCl, NaH_2_PO_4_, HCl, NaOH, NaCl), potassium hydroxide, potassium hydrogen phthalate and tris(hydroxymethyl)aminomethane were purchased from Sigma-Aldrich Co. LLC. (St. Louis, MO, USA). Prisma^HT^ buffer was obtained from Pion Inc. (Billerica, MA, USA). 0.5 M potassium hydroxide and 0.5 M hydrochloric acid volumetric solutions were standardized by titration against primary standards potassium hydrogen phthalate and tris(hydroxymethyl)aminomethane, respectively. Deuterium oxide (D_2_O) and methanol (MeOH) were obtained from Merck Ltd. (Darmstadt, Germany).

### 2.2. pKa Measurement by UV and NMR

#### 2.2.1. UV/pH Titrations in Aqueous Solutions

The p*K*_a_ values of telmisartan were determined by UV-pH titration using the D-PAS™ ultra-violet spectrophotometer attached to a GLpKa instrument (Sirius Analytical Instruments Ltd., Forest Row, UK) [37,38]. First, a stock solution of TEL was prepared in methanol (2 mg/mL). After 100 times dilution by 0.15 M KCl solution, the samples were titrated from pH 10 to pH 1.8 under nitrogen atmosphere at constant ionic strength (I = 0.15 M KCl) and temperature (t = 25.0 ± 0.5 °C or 37.0 ± 0.5 °C).

Spectra were registered in the 200–700 nm region. The p*K*_a_ values were calculated by RefinementPro^TM^ software (Sirius Analytical Instruments Ltd., Forest Row, UK).

#### 2.2.2. UV/pH Titrations in Methanol-Water Mixtures

The cosolvent dissociation constants (p_s_*K*_a_ values) were determined in various MeOH-water mixtures between 8–34% (*w*/*w*) under the same experimental conditions as in aqueous medium.

The aqueous p*K*_a_ values were calculated from the measured p_s_*K*_a_ values in methanol-water solutions using the Yasuda–Shedlovsky extrapolation method.

The Yasuda–Shedlovsky procedure is based on the linear relation between p_s_*K*_a_ and the dielectric constant (*ε*) of the cosolvent mixture:

(1) $$p_{s}K_{a}+\log\left[H_{2}O\right]=\frac{a}{\varepsilon }+b$$

where *ε* is the dielectric constant, and log[H_2_O] is the molar water concentration of the given methanol-water mixture [14].

##### H NMR/pH Titrations

All NMR measurements were performed on a Varian 600 MHz spectrometer (Varian Inc., Palo Alto, CA, USA) with a dual 5 mm inverse-detection gradient probehead at 25 °C, constant ionic strength (*I* = 0.15 M) and using 5 *v*/*v* % D_2_O–95 *v*/*v* % H_2_O as solvent. The spectra were referenced to internal DSS (sodium 3-(trimethylsilyl)-1-propanesulfonate) as chemical shift reference. The pH of the samples (600 μL)were adjusted with concentrated HCl and NaOH and determined by in situ internal indicator molecules optimized for NMR [39,40]. The concentration of telmisartan was below 1 mM during in the titration solution, due to its poor solubility, but all the peaks were still clearly visible. The water resonance was diminished by a double pulse field gradient spin echo sequence (number of transients = 16, number of points = 16,384, acquisition time = 3.33 s, relaxation delay = 1.5 s).

In order to obtain the acid dissociation constants from the chemical shifts–pH profiles, the software Origin Pro 8 (OriginLab Corp., Northampton, MA, USA) was used to perform non-linear regression with the following function [41]:

(2) $$\delta_{\mathrm{obs}}\left(\mathrm{pH}\right)=\frac{\delta_{X^{-}}+\sum_{i=1}^{n}\delta_{H_{i}X^{i-1}}\times 10^{\log\beta_{i}-i\times \mathrm{pH}}}{1+\sum_{i=1}^{n}10^{\log\beta_{i}-i\times \mathrm{pH}}}$$

where $\delta_{X^{-}}$ is the chemical shift of the unprotonated ligand (X^−^), $\delta_{H_{i}X^{i-1}}$ values stand for the chemical shifts of successively protonated species of X^−^, n is the maximum number of protons that can bind to the unprotonated ligand, *β* is the cumulative macroconstant. The standard deviations of log*β* values from the regression analyses were used to calculate the Gaussian propagation of uncertainty for the other equilibrium constants.

### 2.3. Kinetic Solubility Measurement

The kinetic solubility measurements of TEL were determined using µDISS™ profiler (Pion Inc., Billerica, MA, USA). The method was described earlier by Kadar et al. [13].

### 2.4. Thermodynamic Solubility Measurement

The methods for thermodynamic solubility measurements can be found in Baka et al. (measured amount of TEL: 1 mg/mL, 37 °C, 6 h strirring, 18 h sedimentation) [17]. During the thermodynamic solubility measurements in Prisma buffers the concentration of the API was determined without filtering the solutions by the Rainbow Dynamic Dissolution Monitor instrument (Pion Inc., Billerica, MA, USA). In the case of solubility measurements in distilled water, the concentration of the API after filtration with PVDF membrane (0.22 µm) was determined by Jasco V-550 UV–vis spectrophotometer (Jasco Inc., Easton, MD, USA).

### 2.5. XRD

X-ray powder diffraction (XRD) measurements were performed by X’pert Pro MPD X-ray diffractometer (PANalytical B.v., Armelo, The Netherlands) [1,13].

### 2.6. Analysis of Solubility-pH Data

For the analysis of solubility-pH profile, the *p*DISOL-X^TM^ program (in-ADME Research, NY, USA) was used [24,26]. The mathematical approach used in the program for log S-pH simulation-refinement has been described by Völgyi et al. [38]. The program has been applied in several other recent studies [23,42,43,44,45,46].

### 2.7. Mass Spectrometry

An ESI- triple quadrupole-MS/MS instrument (4000 Q TRAP) from Applied Biosystems (Foster City, CA, USA) was used for the MS and MS/MS measurements. Compounds were analyzed in the positive mode, and the settings were ion spray voltage 4500 V, ion source gas (N_2_) 16 arbitrary units (a.u.), curtain gas (N_2_) 10 a.u., declustering potential 20 V, and entrance potential 10 V. For MS/MS experiments, the settings were collision gas (N_2_) medium, and collision energy 51 V. Saturated samples were infused at a rate of 6 μL/min using Harvard Apparatus Syringe pump (Holliston, MA, USA). Analyst 1.4.2. software from Applied Biosystems (Foster City, CA, USA) was used for data acquisition and processing.

### 2.8. Computational Methods for Studying Dimerization

Theoretical calculations were performed by using the Gaussian 09 program package [47]. Preliminary optimizations were carried out by using the PM6 semiempirical method [48]. The optimized structures were further refined by using the M06-2X global hybrid density functional [49] in combination with the 6-31G(d) basis set. Solvent effects were considered by using the conductor-like polarizable continuum model (CPCM) with the parameters of water [50]. Normal mode analysis was also carried out on each structure to verify their position on the potential energy surface.

## 3. Results and Discussion

### 3.1. pK_a_ Determination by UV, Potentiometric and NMR Methods

TEL is a polyfunctional, ampholyte molecule, containing two basic benzimidazole rings and an acidic carboxyl group. The acid-base chemistry of the molecule was studied by various methods. Potentiometry, UV-pH titrations, as well as ^1^H NMR-pH titration were used for the determination of ionization macroconstants. Due to the very poor water solubility of TEL, potentiometry in aqueous solution could not be applied due to the precipitation of the compound. However, TEL possesses pH-dependent UV absorption due to the chromophores in the proximity of the ionizable groups, and therefore the p*K*_a_ values at 25.0 ± 0.5 °C and at 0.15 M ionic strength were determined by UV-pH titration and were as follows: p*K*_a1_ =3.03 ± 0.04, p*K*_a2_ = 4.22 ± 0.09 and p*K*_a3_ = 6.08 ± 0.07.

The measurements were repeated in the same medium, but at biorelevant temperature (37.0 ± 0.5 °C) and only a small deviance was found (p*K*_a1_ = 3.04 ± 0.03, p*K*_a2_ = 4.08 ± 0.02 and p*K*_a3_ = 5.91 ± 0.05), as expected from thermodynamic considerations.

UV-pH titration in methanol-water mixtures was also performed to characterize the ionization pathway of the molecule. In methanol-water mixtures, cosolvent ionization macroconstants (p_s_*K*_a_) can be determined. The dielectric constant of methanol-water mixtures is lower than that of water, which influences the ionization equilibria. For this reason, the p_s_*K*_a_ values of acids are higher, while those of bases are lower than their corresponding aqueous values. The aqueous p*K*_a_ values from the measured cosolvent p_s_*K*_a_ values were obtained by Yasuda–Shedlovsky extrapolation (p*K*_a1_ = 3.03 ± 0.06, p*K*_a2_ = 4.19 ± 0.07 and p*K*_a3_ = 6.14 ± 0.10). The advantage of this cosolvent method is that it allows for assigning p*K*_a_ values to the acidic and basic moieties of the molecule. The slopes of the Yasuda–Shedlovsky equations indicate the acid-base property of the functional group. The basic functional groups have negative slopes, while for the acidic functions the slopes are typically positive. Based on these data, the p*K*_a2_ and p*K*_a3_ values mainly characterize the ionization of the two benzimidazole rings, while the p*K*_a1_ constant describes the ionization of the carboxyl group.

Since the ionization of the three functional groups of TEL is highly overlapping, for the exact characterization of its acid/base property at submolecular level the protonation microconstants would be required [51]. However, the determination of microconstants is hindered by the close proximity of ionizable groups. Therefore, all measured p*K*_a_ values are protonation macroconstants in this study.

**Figure 1:**
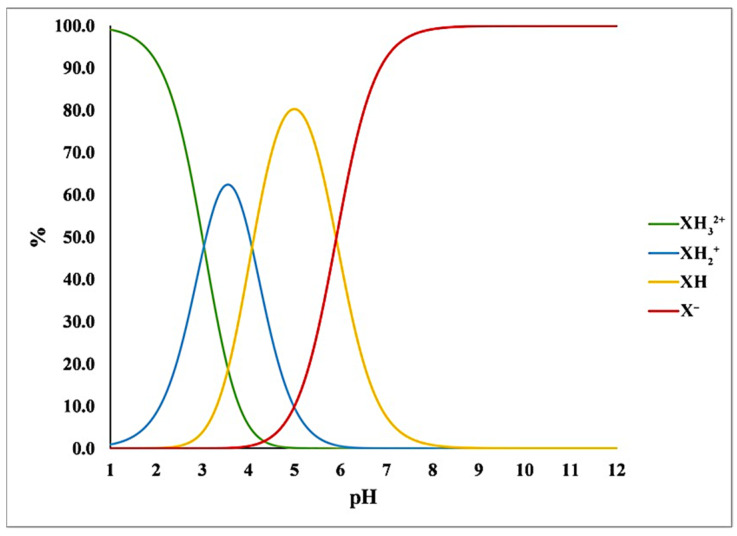
The pH-dependent distribution of telmisartan macrospecies.

The percentage of various protonated macrospecies can be calculated for any arbitrary pH value using the measured p*K*_a_ values at 37 °C. The distribution of each macrospecies as a function of pH is shown in Figure 1. At gastric pH (pH 1–2), the dication (XH_3_^2+^) form predominates, while at intestinal pH (pH 5–6.5) the monoprotonated (XH) and the unprotonated anionic (X^−^) forms are the dominant species. At the pH of plasma (pH 7.4), TEL exists mainly in anionic (X^−^) form.

**Figure 2:**
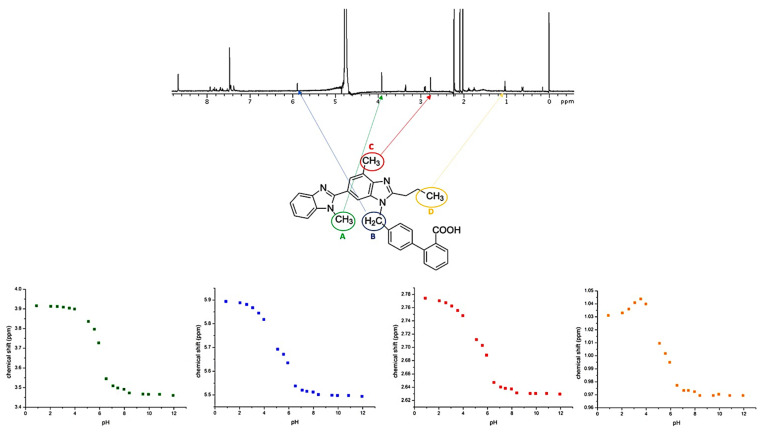
^1^H NMR spectrum(**top**) and ^1^H NMR pH-titration curves (**bottom**) of telmisartan.

Evaluation of the ionization constants from ^1^H NMR-pH titration curves was based on the principle that non-exchanging NMR nuclei near the basic site sense different electronic environments upon ionization and change their chemical shift accordingly. As there are numerous chemically different ^1^H nuclei in the TEL molecule and the chemical shift of these signals changes due to ionization, in the ^1^H NMR spectra most of the signals overlapped (especially those of the aromatic rings). Thus, only four signals of chemically different protons (H_A_, H_B_, H_C_, H_D_, see Figure 2 top) that could be observed provided useful results. The NMR spectra of the fully protonated (pH = 1.75) and deprotonated (pH = 7.75) ionization form of TEL can be found in Supplementary Materials (Figures S1 and S2). The ionization macroconstants of TEL were determined by investigating the chemical shift changes of the above-mentioned protons in ^1^H NMR-pH titrations (Figure 2 bottom). The resulting macroconstant values based on non-linear regression analysis are p*K*_a1_ = 3.21 ± 0.14; p*K*_a2_ = 4.28 ± 0.07; p*K*_a3_ = 6.08 ± 0.04.

It is notable that the limitation of NMR spectroscopy for the titration of TEL is the poor solubility of the compound and the overlapping signals. However, the macroconstant values determined by ^1^H NMR-pH titration and UV-pH titration (which is a reliable method in case of low analyte concentration) are in good agreement (Table 1).

Since the effect of ionization on the chemical shifts diminishes along with the increasing distance from the site of ionization, based on the ^1^H NMR titration data we can obtain information about the dominant ionization pathway. As the H_A_ hydrogen of the methyl group bonded to one of the benzimidazole nitrogen shows the biggest chemical shift change between pH 5 and 7, the third ionization step occurs dominantly on the benzimidazole structure farther from the carboxyl group. The H_B_ hydrogen signal changes primarily between pH 3.5 and 5, while the titration curve of the H_C_ protons shows the first two ionization steps slightly. Based on these observations, the second ionization step can be assigned to the benzimidazole group closer to the carboxyl group. The ionization of the carboxyl group influences the electronic environment of the sterically proximate methyl protons (H_D_) of the propyl group connected to the benzimidazole ring.

The assignation of the spectra is based on the multiplicity and the integral of the signals and the evidence that a heteroatom in three covalent bonds results in significantly higher chemical shift values. Thus, further measurements were not required.

### 3.2. Kinetic Solubility Measurements

**Figure 3:**
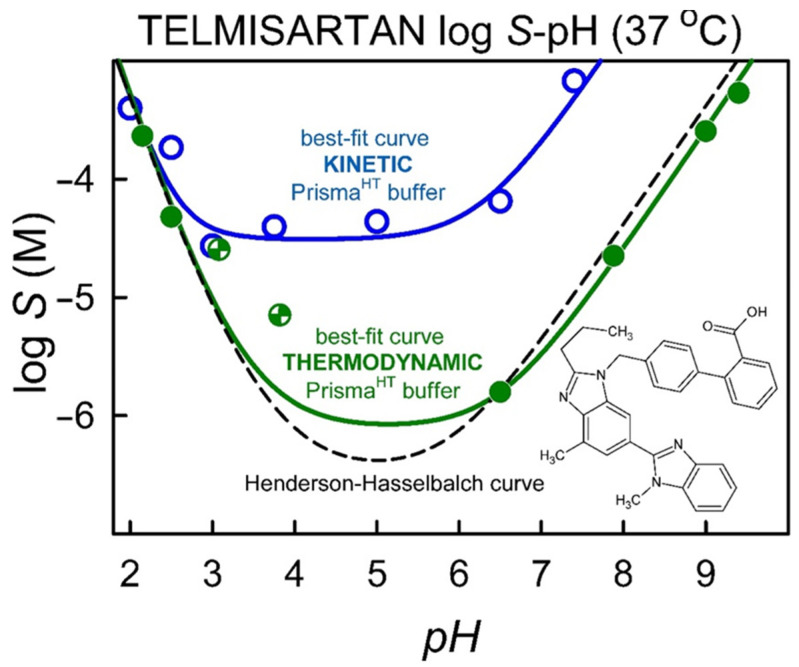
Thermodynamic and kinetic solubility-pH profile of Telmisartan in Prisma^HT^ buffer at 37 °C.

The results of kinetic solubility measurements can be seen in Figure 3. As expected from the API’s structure and p*K*_a_ values, the kinetic solubility is highly dependent on the pH of the aqueous media: solubility is minimal in the pH range 3–6 and reaches higher values in more acidic or basic media. Because of the high concentration values it could not be measured with the described UV method (see Section 2) above pH 7.4.

Comparing the results to the thermodynamic solubilities, a near two-magnitude difference could be seen between the thermodynamic and kinetic solubility data from pH = 3 to pH = 8 (where the zwitterionic form of the API is present). In contrast, no significant difference was detected at more acidic pHs. These results indicate that TEL’s ability to form a supersaturated solution is highly pH-dependent.

### 3.3. Thermodynamic Solubility and XRD Results

The average values of the equilibrium solubility results obtained at 11 different pHs are shown in Table 2. The relative standard deviation (coefficient of variation) was found to be in the range of 1–15%.

**Figure 4:**
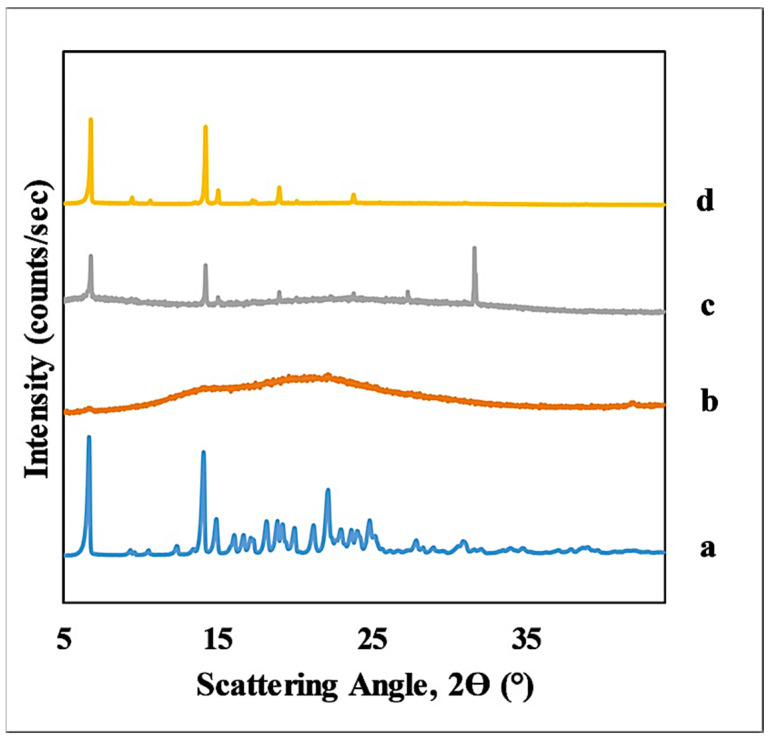
X-ray diffractograms of pure TEL and the solid phase isolated from the solubility suspension from the measurement at different pH: (**a**) TEL_crystalline, (**b**) 1M NaOH, (**c**) pH 9.5–10, (**d**) pH 1.6–9.

XRD was used to observe the changes in the crystalline structure of TEL induced by solubility measurements. Figure 4 shows the diffractograms of the solid phase isolated from the suspension of the solubility measurement compared to that of the crystalline API. In the case of the crystalline API and the isolated solid phase from solubility measurements between pH 1.6 and 9 (Figure 4a,d), the characteristic peaks of the crystalline drug were clearly observed. Additionally, the amorphous background is observed in the diffractograms of the pH 9.5–10 solid phases. At the same time, the characteristic peaks of the API are also apparent. Meanwhile, in the case of the solid phase from the 1 M NaOH measurement (Figure 4b), no diffraction peaks were seen, only a diffuse background scattering (halo) as proof of the amorphous nature of the sample. It might be surprising at first that an amorphous form of the sodium salt is in equilibrium with the aqueous phase. However, the fact that this drug is marketed as an amorphous sodium salt also shows that this solid form is very stable [52]. A characteristic peak at 32 degrees 2Θ is visible in the Figure 4c diffractogram. This peak was identified by measuring some samples up to 94 degrees 2Θ. Based on comparison with the Cambridge database, this peak belongs to NaCl in solution.

In order to eliminate the effects of the buffer components, the thermodynamic solubility measurements were also performed in distilled water. The deviation of the experimental data from the theoretical Henderson–Hasselbalch curve was noted. The average values of the equilibrium solubility results obtained at 6 different pHs are shown in Table 3.

Comparing the results of the thermodynamic solubilities in Prisma buffer and distilled water, no major difference could be seen between the different solvents.

**Figure 5:**
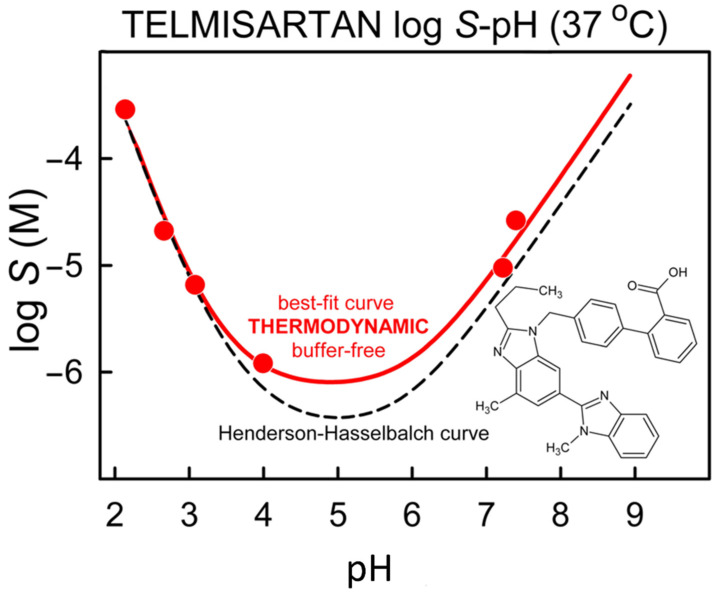
log S-pH profile of Telmisartan in distilled water at 37 °C.

The thermodynamic solubility-pH data in distilled water were analyzed with the pDISOL-X software [24,26]. The calculated intrinsic solubility of TEL is logS_0_ = −6.52 ± 0.08 and the dimerization constants are logK_22_ = 6.35 ± 0.47, logK_21_ = 6.48 ± 0.19. The slight deviation of the experimental data from the theoretical Henderson–Hasselbalch curve (Figure 5) can be thus explained by the existence of zwitterionic aggregates (XH.XH and XH.X^−^) in the solution, according to the software.

Interestingly the pH range where high supersaturation is detected (see kinetic solubility data) is the same where the zwitterionic form of the API is present and zwitterionic aggregates can be expected, based on the deviation from HH equation. Based on these data and observations we formed the hypothesis that the formation of zwitterionic aggregates may enable the API to reach such high supersaturation in aqueous solution.

### 3.4. Mass Spectrometry

**Figure 6:**
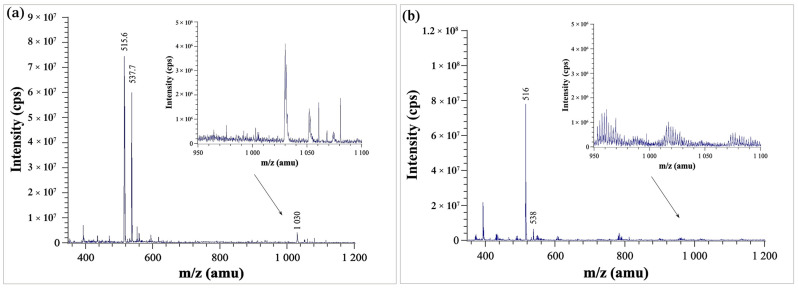
(**a**) MS spectra of buffer-free saturated solution at pH 3–8, (**b**) MS spectra of buffer-free saturated solution at pH = 10.

Mass spectrometry is an appropriate technique to examine the aggregation phenomena according to the literature [25]. Therefore, it was chosen to confirm the aggregation of TEL in solution in the pH range of 3–8. An example of MS spectra of TEL containing solutions is shown in Figure 6a.

The most intense peak in the MS spectra was the one belonging to [Y + H]^+^ = 515.6 Da, where Y is the molecular mass of the TEL unit (514.62 g/mol). The adduct of sodium and TEL was also observed ([Y + Na]^+^ = 537.7 Da), and with lower intensity a peak corresponding to a dimeric structure ([2 Y + H]^+^ = 1030.0 Da) was detected. Neither trimers nor higher forms of aggregates were detected.

While in the pH range of 3–8 the peak corresponding to the dimeric structure could be detected throughout the whole pH range, at higher pHs (e.g., pH 10) no dimerization could be observed (at lower pHs the MS measurement was not technically possible).

This observation supports our hypothesis that in aqueous solution the formation of zwitterionic agglomerates can be expected in the pH range 3–8. It also supports the idea that TEL’s ability to reach such high supersaturation in solution seems to be due to the dimerization in solution. Since the dimers only form in a specific pH range (3–8), they most likely exist due to the aggregation of the zwitterionic form of TEL (Figure 1).

### 3.5. Studying Dimerization by Computational Chemistry

**Figure 7:**
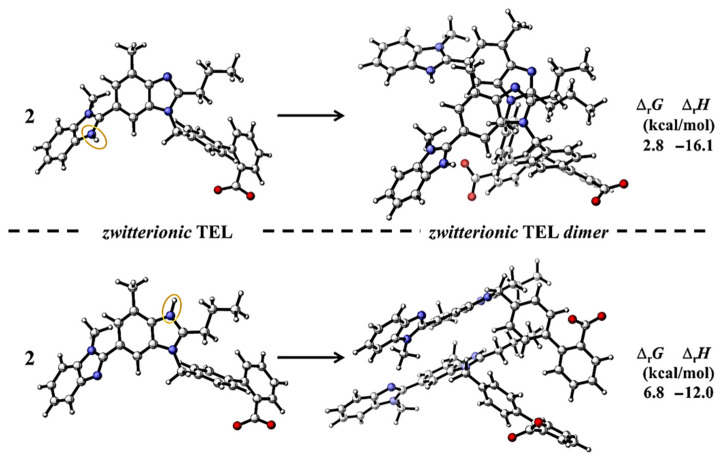
Dimerization of zwitterionic telmisartan along with the corresponding reaction Gibbs free energy (Δ_r_*G*) and enthalpy (Δ_r_*H*) values in kcal/mol. The structures have been computed at the M06-2X/6-31G(d) level of theory. The effect of water was mimicked by using the CPCM implicit solvent model. Orange ellipsoids indicate the protonation sites.

To further support the mass spectrometry results, the potential dimerization processes of zwitterionic TEL structures have been considered and studied by computational chemistry methods. The optimizations were performed by using the PM6 semiempirical method in combination with the CPCM implicit solvent model to mimic the effect of water, and then refined by using the M06-2X/6-31G(d) level of theory (Figure 7).

Two different zwitterionic TEL structures are considered, depending on which methyl benzimidazole group is protonated. Furthermore, two dimers including two of only one or the other zwitterionic TEL structures computed. From a structural point of view, attractive stacking interactions are dominant in the formed zwitterionic dimers and both, parallel displaced (between the middle benzimidazole units) and T-shaped stacking (between the benzoic acid groups) types can be identified (Figure 7). Thus, in case of terminal protonation (Figure 7, top) the dimer formed in a way that lipophilic groups are facing towards each other, while hydrophilic groups are facing towards the aqueous outer space. This agrees well with the hypothesis that the formation of dimers might play an important role in the supersaturation of TEL in aqueous media. In terms of the thermodynamic properties of the process, the reaction Gibbs free energy (Δ_r_*G*) is 2.8 kcal/mol, while the corresponding dimerization enthalpy (Δ_r_*H*) is −16.1 kcal/mol when the terminal benzimidazole unit of the telmisartan is protonated and the dimer is formed from that structure (Figure 7, top). The negative enthalpy indicates that the dimerization is energetically favored, although considering the reaction’s Gibbs free energy it is not a spontaneous process. This computational results agree with the experimental observations, that supersaturation needs to be induced by the pH-shift method, but the supersaturated solution itself is fairly stable [53].

In case of the protonation of the middle benzimidazole unit, the dimerization is less favored in aqueous environment by ~4 kcal/mol (Figure 7, bottom). This is in fairly good agreement with the experimental findings of the NMR-pH titration which indicated that the terminal protonation is the dominant ionization pathway.

## 4. Conclusions

This work describes the pH-dependent solubility and supersaturation of TEL based on thermodynamic and kinetic solubility measurements carried out with state-of-the-art experimental techniques. The collected data show that TEL is highly capable of forming supersaturation in solution in the pH range 3–8, while at lower or higher pHs no supersaturation takes place. Experimental data of thermodynamic solubility shows a slight deiation from the Henderson–Hasselbalch theoretical curve, which is explained by the presence zwitterionic aggregates in solution. Based on the measured p*K*_a_ values and distribution of microspecies, it can be concluded that the pH range (3–8) where high supersaturation-capacity is observed is the same where the zwitterionic (non-ionic) form of TEL is present. The dimerization of the API was confirmed experimentally in the pH range of 3 to 8, using the ESI-MS technique, substantiating the existence of zwitterionic aggregation. Dimerization of zwitterionic structures was also studied by using computational chemistry tools to get additional details about the role of dimers in the supersaturation of TEL. This study contributes to the better understanding of supersaturation at molecular level and provides an example of the importance of pH-dependent (kinetic and thermodynamic) solubility measurement in case of BCS II. compounds.

## Figures and Tables

**Table 1:** Ionization macroconstants of Telmisartan measured by different methods.

Method	Ionization Macroconstansts		
	p*K*_a1_ ± SD (Carboxyl Group)	p*K*_a2_ ± SD (Middle Benzimidazole Ring)	p*K*_a3_ ± SD (Terminal Benzimidazole Ring)
**UV/pH titration in aqueous medium (37 °C)**	3.04 ± 0.03	4.08 ± 0.02	5.91 ± 0.05
**UV/pH titration in aqueous medium (25 °C)**	3.03 ± 0.04	4.22 ± 0.09	6.08 ± 0.07
**UV/pH titration in MeOH-water mixture (25 °C)**	3.03 ± 0.06	4.19 ± 0.07	6.14 ± 0.10
**NMR-pH titration (25 °C)**	3.21 ± 0.14	4.28 ± 0.07	6.08 ± 0.04

**Table 2:** Equilibrium solubility of Telmisartan in Prisma^HT^ buffer.

pH	S ± SD (µg/mL)	logS ± SD (M)	XRD Characterization
**1.60**	150.00 ± 38.00	−3.54 ± 0.05	Crystalline
**2.15**	119.01 ± 11.79	−3.64 ± 0.04	
**2.50**	16.99 ± 0.72	−4.32 ± 0.02	
**3.08**	13.05 ± 1.73	−4.60 ± 0.06	
**3.82**	3.62 ± 0.59	−5.15 ± 0.13	
**6.50**	0.79 ± 0.16	−5.80 ± 0.15	
**7.88**	11.54 ± 0.26	−4.65 ± 0.01	
**9.00**	130.41 ± 2.73	−3.60 ± 0.01	amorphous background
**9.40**	274.95 ± 9.40	−3.27 ± 0.02	
**9.79**	1472.67 ± 254.55	−2.54 ± 0.07	
**11.74**	2250.10 ± 144.82	−2.36 ± 0.03	Amorphous

**Table 3:** Equilibrium solubility of Telmisartan in distilled water.

pH	S ± SD (µg/mL)	logS ± SD (M)
**2.16**	147.72 ± 5.48	−3.54 ± 0.02
**2.66**	10.83 ± 0.53	−4.68 ± 0.02
**3.08**	3.4 ± 0.49	−5.18 ± 0.06
**3.98**	0.36 ± 0.12	−6.16 ± 0.15
**7.22**	3.65 ± 0.11	−5.15 ± 0.01
**7.4**	13.80 ± 2.81	−4.57 ± 0.09

## Data Availability

All data can be provided by the authors upon request. No publicly accessable archive storage is available.

## Supplementary Materials

The following supporting information can be downloaded at: https://www.mdpi.com/article/10.3390/pharmaceutics14081635/s1, Figure S1: NMR spectrum of TEL at pH 1.75 (please note that for better illustration only the aliphatic and aromatic region of the spectrum is depicted); Figure S2: NMR spectrum of TEL at pH 7.75 (please note that for better illustration only the aliphatic and aromatic region of the spectrum is depicted).
