# Supplementary material for: Understanding the pH Dependence of Supersaturation State—A Case Study of Telmisartan
Source: Pharmaceutics. 2022 Aug 5;14(8):1635. doi: 10.3390/pharmaceutics14081635 (PMC9412861; doi:10.3390/pharmaceutics14081635)
Supplement: Supplementary file 1 [file pharmaceutics-14-01635-s001.zip › pharmaceutics-1840889-supplementary.pdf]

Supplementary Materials: Understanding the pH dependence of Supersaturation State—A Case Study of Telmisartan

Szabina Kádár, Dóra Csicsák, Petra Tőzsér, Attila Farkas, Tamás Pálla, Arash Mirzahosseini, Blanka Tóth, Gergő Tóth, Béla Fiser, Péter Horváth, János Madarász, Alex Avdeef, Krisztina Takács-Novák, Bálint Sinkó, Enikő Borbás and Gergely Völgyi

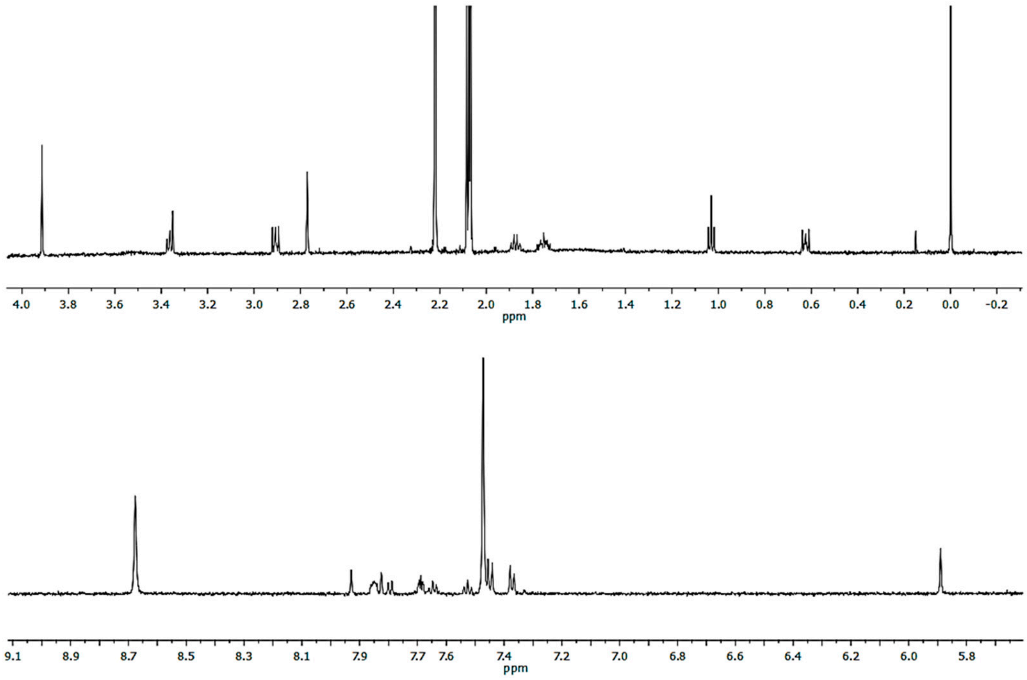

Figure S1. NMR spectrum of TEL at pH 1.75 (please note that for better illustration only the aliphatic and aromatic region of the spectrum is depicted).

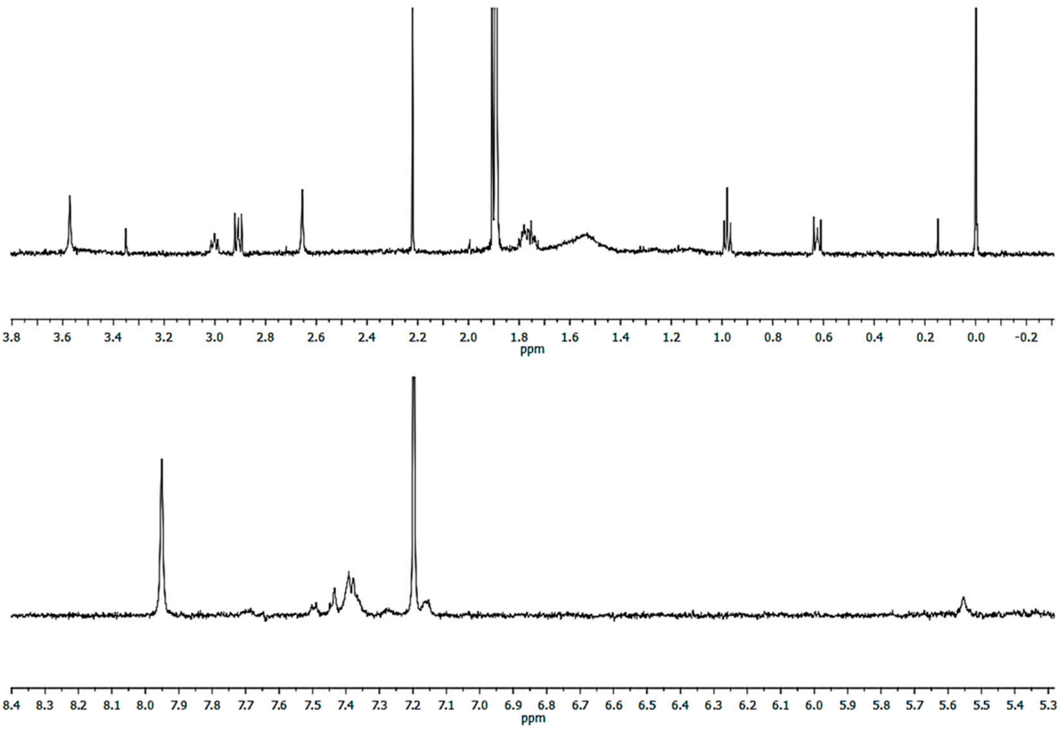

Figure S2. NMR spectrum of TEL at pH 7.75 (please note that for better illustration only the aliphatic and aromatic region of the spectrum is depicted).
